# Effects of hypertonic saline (7.5%) on lactate clearance compared with normal saline (0.9%) after on pump cardiovascular surgery. a randomized controlled trial

**DOI:** 10.1186/2197-425X-3-S1-A953

**Published:** 2015-10-01

**Authors:** LH Atehortua Lopez, R Mendoza, A Urrego, JF Escobar, FA Jaimes Barragan

**Affiliations:** Hospital Universitario de Sanvicente Fundación, Universidad de Antioquia, UCI Cardiovascular, Medellin, Colombia; Universidad de Antioquia, Medicina Critica y Cuidado Intensivo, Medellin, Colombia; Universidad de Antioquia, Medicina Critica y Cuidado Intensivo, Medellín, Colombia; Unversidad de Antioquia, Medicina Interna, Medellín, Colombia

## Introduction

Cardiovascular care after the CPB pump often require an exhaustive reanimation^,^because reduces the intravascular oncotic pressure and increases the capillary permeability.The crystalloids might increase edema and is well known that fluid overload increases mortality.The colloids have not proved a better outcome.A option is to use hypertonic solutions.Lactate is reliable for monitoring adequate tissue perfusion during extracorporeal life support and is strongly predictive of mortality.

## Objectives

To compare the effect of the 7.5% hypertonic saline (HS) with the 0.9% normal saline NS on decreasing the level of lactate in patients submitted to CPB after 24 hours in an ICU as well as on the effect over hemodynamic variables.

## Methods and Design

One centred,parallel, randomized controlled double blind trial. 102 Patients 18 years or older with coronary artery disease and heart valve disease who underwent coronary artery bypass graft or heart valve replacement surgery with CPB.Patients were randomized to receive 4 mL/kg of predicted body weight of HS (HS group) or an equal volume of NS (NS group)intravenous for 30 minutes starting after admission of the patient to the ICU.We measured with a pulmonary artery catheter the CI,CVP and PWP at 0 hours, 30 minutes, 4 hours, 6 hours, 12 hours and 24 hours after the admission in the ICU, it was obtained also mixed oxygen saturation, arterial blood gases at 0 hours, 6 hours, 12 hours and 24 hours after the admission of the patient in the ICU.The primary outcome was to achieve in HS group a higher lactate clearance than in the NS group after 24 hours of admission in the ICU. The secondary outcome was to improve the hemodynamic variables in the HS group compared with the NS group.

## Results

Between April 18, 2013, and January 20, 2015, a total of 102 patients were included in the study and randomly assigned to HS (51 patients) or NS (51 patients) groups.The mean age of the 102 study participants was 59 years (range, 22 to 84); 71 participants (59.7%) were men. After randomization, 11 patients in the HSS group and 11 patients in the NS group did not complete the follow up because they lack of complete data of blood lactate levels during the study. No significant difference was observed between the two study groups with respect to lactate blood levels (Table 3). No significant differences were observed in other secondary outcomes, there was also no difference in mortality and length of stay in the ICU. (Table 2)

## Conclusions

Our study failed to demonstrate a higher lactate clearance with the use of one dose of HS, but there was not a higher incidence of side effects in the HS group.

